# A Rare Case of Recurrent Pneumonia in Heterotaxy Syndrome, Polysplenia/Left Isomerism

**DOI:** 10.7759/cureus.19055

**Published:** 2021-10-26

**Authors:** Ayesha Anwar, Jacob Jubin, Shafi Raza, Zafar K Mirza

**Affiliations:** 1 Department of Medicine, Allama Iqbal Medical College/Jinnah Hospital, Lahore, PAK; 2 Radiology, Olean General Hospital/University at Buffalo, Olean, USA; 3 Hospital Medicine, Olean General Hospital/University at Buffalo, Olean, USA; 4 Gastroenterology and Hepatology, Olean General Hospital/University at Buffalo, Olean, USA

**Keywords:** congenital heart defects, situs ambiguous, immunisation, left atrial isomerism, pneumonia, polysplenia, heterotaxy syndrome

## Abstract

Heterotaxy syndrome (HS) or situs ambiguous refers to the abnormal arrangement of viscera across the body axis, and abnormalities arise depending on the isomerism of the right or left atrial appendage. The cause remains unexplained and is attributed to a combination of genetic mutations and environmental factors. It is a rare condition and may remain undiagnosed for a long time. In this report, we aim to highlight an unusual presentation and aggravation of an infection due to the underlying isomerism of the left atrial appendage. We discuss the case of a female patient who presented with symptoms of fever and cough. The patient underwent prolonged antibiotic treatment, and her recovery was slow. The presence of bilobed lungs, vertical left-bronchus, and polysplenia on CT scan explained the left-sided aspiration pneumonia. The hypofunctioning spleen (polysplenia) caused her to have a weak immunological response, necessitating prolonged antibiotic use. She was followed up over time and had a recurrence of pneumonia within a few months. The condition is associated with high morbidity and mortality, and the role of early diagnosis and reporting to prevent complications is paramount. The recurrent pneumonia observed in the patient also raises questions related to long-term antibiotic use and immunization in the case of polysplenia in this patient population.

## Introduction

Heterotaxy syndrome (HS), also known as situs ambiguous, is a rare, complex birth anomaly that leads to the abnormal positioning of internal thoracoabdominal organs across the left-right axis of the body. The term “heterotaxy” is derived from the Greek words “heteros” meaning “other than” and “taxis” meaning “arrangement” [1]. Situs ambiguous includes manifestations that cannot be classified into either situs solitus or situs inversus. While situs solitus refers to the normal arrangement of these structures, situs inversus is a condition characterized by the mirrored organization of the viscera. It occurs in one out of 10,000 live births, and a large number of these cases result in stillbirths [2]; it is more prevalent in males than females, with a male-to-female ratio of 2:1.

HS is classified based on the characteristics of the spleen or the morphology of atrial appendages. Isomerism refers to the aberrant bilateral symmetry of specific viscera: isomerism of the right atrial appendage (IRAA) or asplenia syndrome, and isomerism of the left atrial appendage (ILAA) or polysplenia [3]. Mutations of genes involved in the lateralization of body organs result in embryonic disruptions in the left-right axis determination. However, due to the heterogeneity in HS, the attempts to identify the precise genetic alterations have remained unyielding to date. The peculiar genotypic and phenotypic aspects of the syndrome result in varying presentations among different individuals. The bronchial-atrial disarrangement in IRAA causes more severe cardiac abnormalities than in ILAA [1]. Moreover, the extracardiac variants involving tracheoesophageal fistula, duodenal atresia, biliary tract malformations, and gastrointestinal malrotations are frequently seen with ILAA [4]. Congenital heart defects lead to high rates of mortality and morbidity. The widespread use and availability of CT scans have made it possible to diagnose heterotaxy as an incidental finding in healthy individuals. This syndrome is associated with diverse anomalies, and delayed diagnosis may lead to a worse prognosis. Prompt diagnosis and early remedial intervention are essential to reduce morbidity and mortality.

Left bronchial isomerism is associated with polysplenia in about three-fifths of the cases, and right bronchial isomerism is found in about seven-tenths of cases with asplenia [5]. Left isomerism is also characterized as bilateral left-sidedness, in which there is the duplication of left-sided viscera such as bilateral pulmonary atria, bilateral bilobed lungs, and bilateral hyperatrial bronchi.

## Case presentation

A previously healthy 34-year-old female with a past medical history of behavioral health problems, including impulse control disorder, bipolar disorder type 1, depression, and post-traumatic stress disorder (PTSD) was referred to the ER by the primary care physician due to the persistent complaint of low-grade fever and cough associated with meals. The patient also complained of nausea and vomiting after meals. There was no history of anorexia, weight loss, headache, dizziness, or blurry vision. She had a history of tracheoesophageal fistula repair and multiple abdominal surgeries for intestinal obstruction. On the basis of suspicion of aspiration pneumonia on a chest X-ray, she was referred to the emergency department. On physical examination in the ER, she was normotensive and afebrile with a blood pressure of 127/81 mmHg, heart rate of 86/minute, temperature of 98.8 °F, and respiratory rate of 16/minute with a saturation of 94% on room air. The lung examination demonstrated normal vesicular breathing with bronchial breathing and decreased air entry on the middle and lower left side of the chest. The rest of the examination was unremarkable.

Investigations

**Figure 1:**
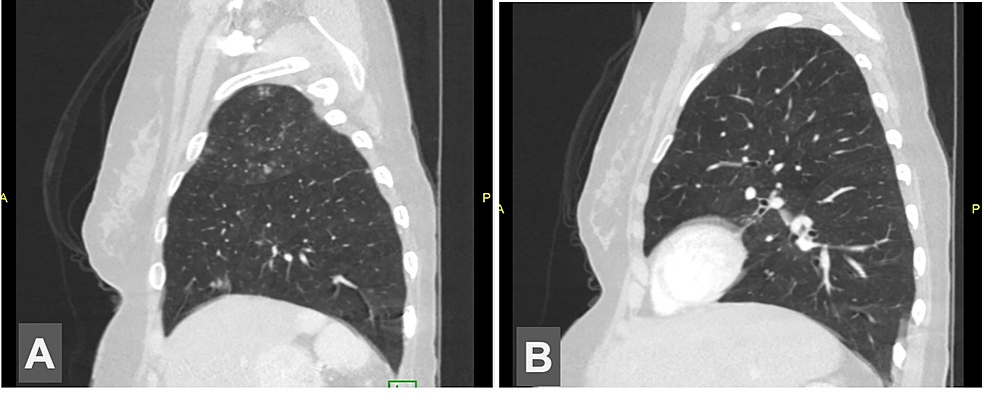
Lateral abdomen CT images A. Bilobar right lung. B. Bilobar left lung CT: computed tomography

**Figure 2:**
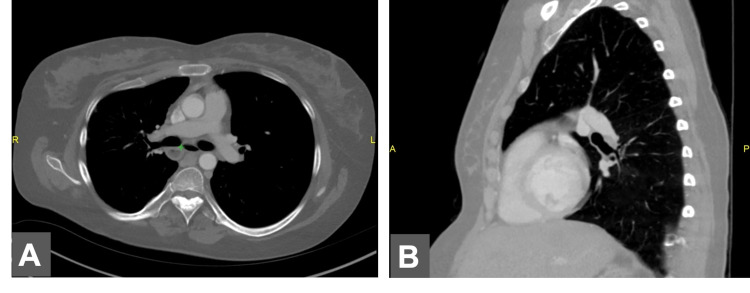
Chest CT images A. Axial chest CT image showing bronchi inferior to the pulmonary artery. B. Lateral chest CT image demonstrating hyperatrial left bronchus CT: computed tomography

**Figure 3:**
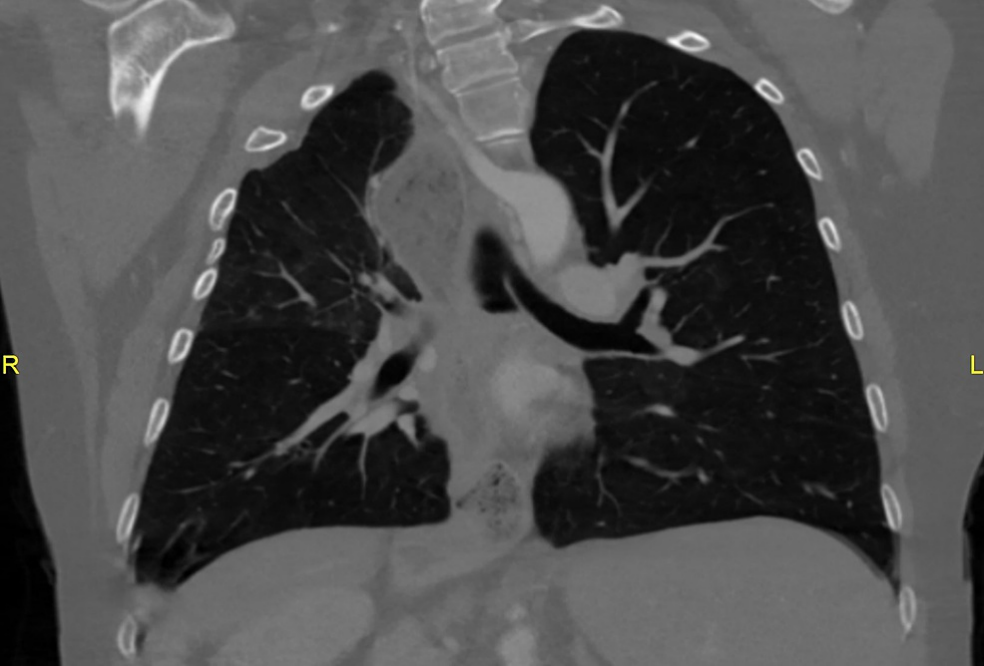
Coronal chest CT image demonstrating left vertical bronchus CT: computed tomography

**Figure 4:**
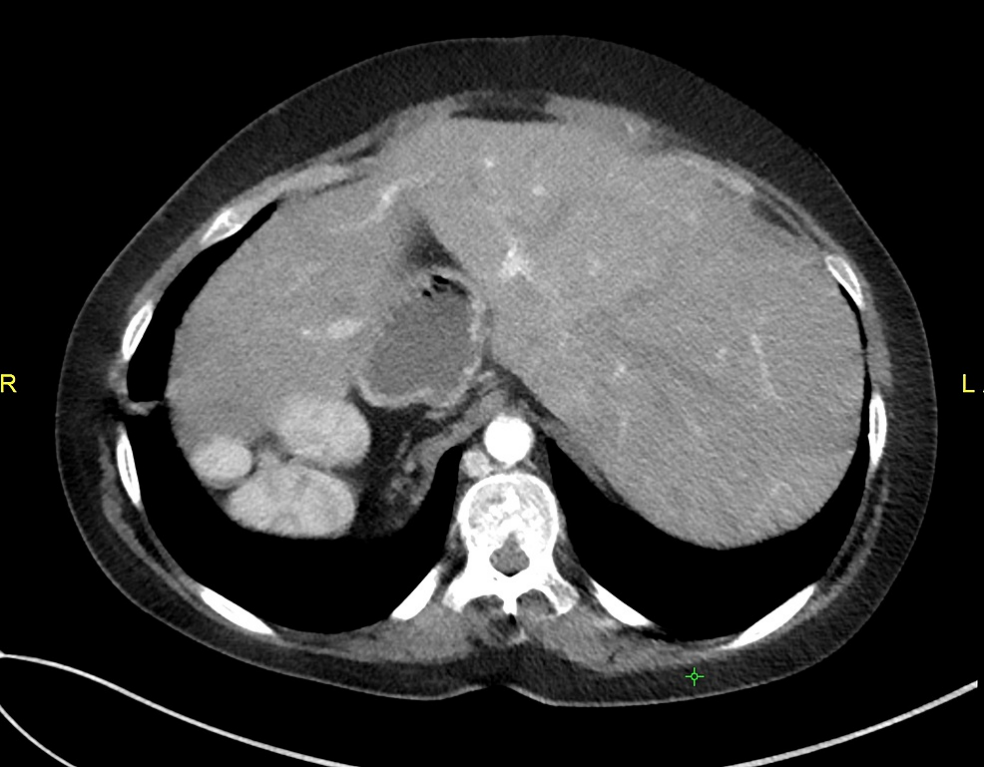
Axial abdominal CT image showing midline liver CT: computed tomography

**Figure 5:**
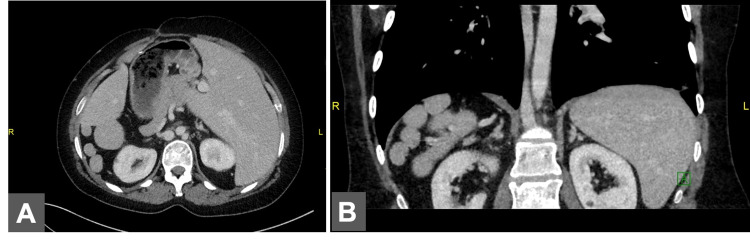
Axial and coronal abdominal CT images A. Axial abdominal CT image. B. Coronal abdominal CT image demonstrating polysplenia CT: computed tomography

**Figure 6:**
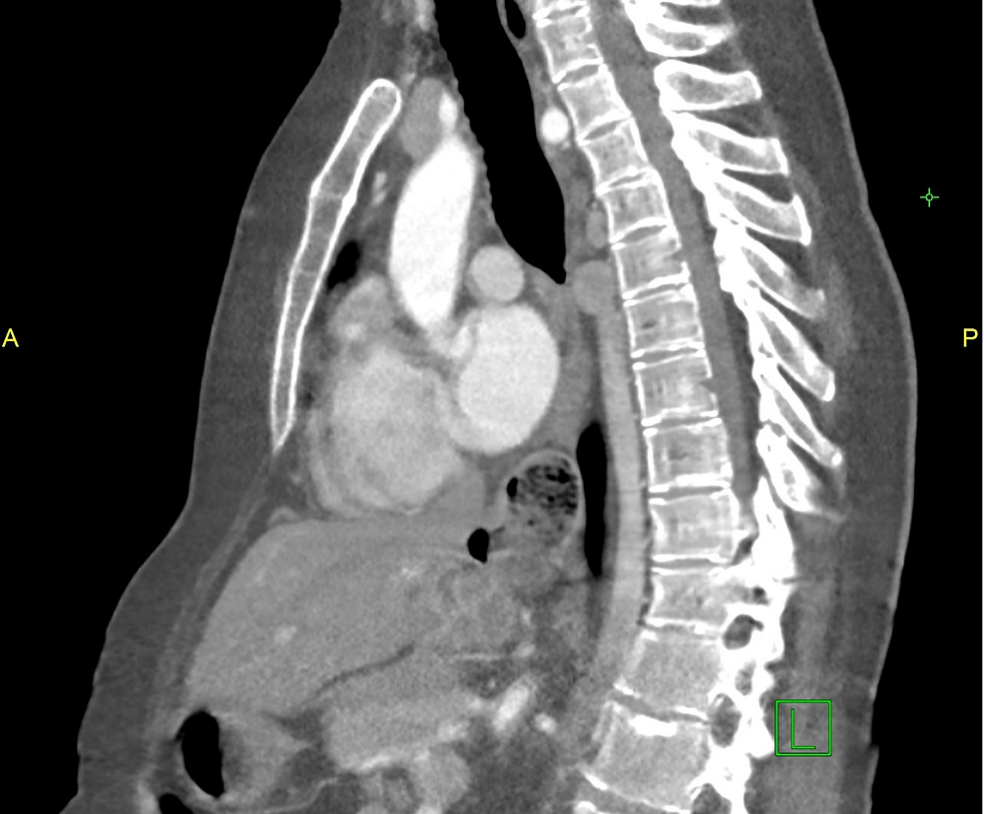
Lateral abdominal CT scan image showing the azygous continuation of inferior vena cava CT: computed tomography

**Figure 7:**
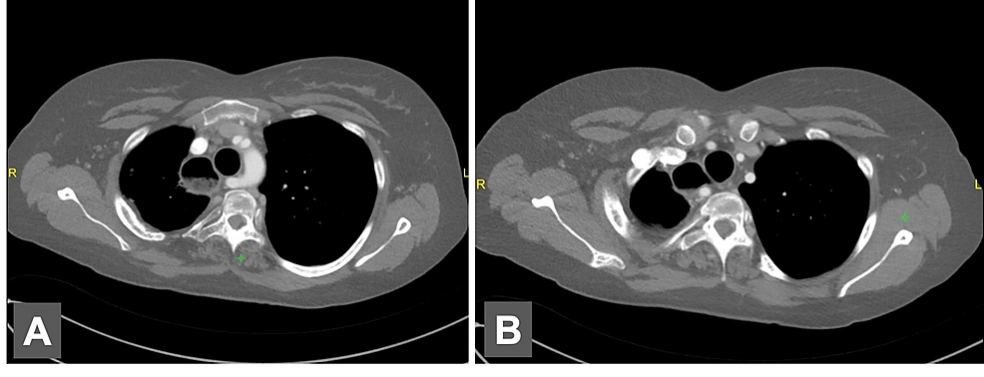
Axial chest CT scan A. Aberrant right subclavian coursing posterior to the esophagus. B. The first branch of the aortic arch is the right common carotid artery, then the left common carotid artery, then the left subclavian, and finally the right aberrant subclavian CT: computed tomography

A CT scan was performed in the ER, which demonstrated two cavitary lesions in the lingula and left lower lobe, raising concerns for aspiration pneumonia. The CT scan also showed bilateral bilobed lungs (Figure 1), heart apex directed towards the left side, left bronchus coursing below the left pulmonary artery (Figure 2), vertical bronchi bilaterally (Figure 3), midline liver (Figure 4), right-sided polysplenia, multiple aberrant nodules of splenic tissue in the right upper quadrant (Figure 5), the azygous continuation of inferior vena cava (Figure 6), and aberrant right subclavian artery (Figure 7), characteristic of heterotaxy. Tuberculosis test (QuantiFERON-TB Gold), beta-D glucan test, and coronavirus disease 2019 (COVID-19) polymerase chain reaction (PCR) nasal swab were sent to rule out tuberculosis, fungal pneumonia, and COVID-19 pneumonia. Blood cultures were also sent.

Treatment

The initial diagnosis was “lung abscess secondary to aspiration pneumonia.” Hence, she was empirically started on piperacillin-tazobactam and vancomycin. A pulmonology consult was obtained and bronchoscopy was planned in case of a worsening condition. The QuantiFERON-TB Gold test, beta-D glucan test, and COVID-19 PCR nasal swab all came back negative. After 48 hours, the creatinine was deranged and dose adjustment for vancomycin was required. After obtaining negative blood cultures on the fifth day, vancomycin was discontinued and piperacillin-tazobactam was continued.

Outcome and follow-up

The patient's cough subsided and creatinine improved. She had no further episodes of fever. She was discharged on day 10 after optimizing her medications. At discharge, she was advised to follow up with pulmonology along with referral to the cardiology clinic for an ECG and echocardiography to perform an assessment of heart disease associated with HS.

Repeat chest X-ray performed one month after the clinical improvement revealed the resolution of cavities. About three months after the initial episode of pneumonia, she again presented to the ER with complaints of cough and lethargy. Chest CT scan demonstrated a new cavitary lesion on the left lower side of the chest. She was managed with the same antibiotics as before and discharged after seven days. Currently, she is doing better and awaiting cardiac workups such as ECG and echocardiogram.

## Discussion

HS is linked with a higher risk of community-acquired severe bacterial infections and increased mortality rate, regardless of the presence or absence of the spleen [6]. It is clear that the absence of splenic function due to asplenia in right atrial isomerism (RAI) predisposes to a higher risk of bacteremia. However, data regarding infection risk in left atrial isomerism (LAI) with polysplenia (qualitative dysfunction of the spleen) is scarce and there is no agreement on the ways to evaluate the splenic function in polysplenia. A systematic review on the incidence of HS associated with bacteremia in children concluded that 79% of cases of bacteremia occurred in HS with anatomic asplenia and 21% in polysplenia. The mortality rate was similar in both groups; 68% in asplenia vs. 60% in polysplenia [7]. This highlights that the hyposplenism in either of the settings carries a higher risk for infections. Moreover, the guidelines for the assessment and antibiotic management for sepsis or infections associated with HS are lacking.

In this patient, the occurrence of recurrent aspiration pneumonia suggests that the causative factor was polysplenia or the presence of multiple nodules of the spleen, which is associated with abnormal splenic function. This suggests that the hypofunctioning spleen or functioning hyposplenism in such patients makes them more susceptible to infections due to their weak immunological response. Unrecognized ciliary dysfunction causing poor secretion clearance also contributes to it. Moreover, the presence of bilobed lungs and vertical left bronchus (similar to the right) gives an equal predisposition to left-sided aspiration pneumonia.

Recurrent episodes of pneumonia in our young patient suggested the presence of impaired immune response and ciliary dysfunction. The cause remains the absence of IgM memory B cells predisposing individuals to repeated infections with encapsulated organisms like *Streptococcus pneumoniae*, *Neisseria meningitidis*, and *Haemophilus influenzae* in both splenectomized individuals and patients with intact spleen [8]. The studies have demonstrated that the mean IgM memory B cells in HS patients are low and it correlates with the risk of severe bacterial infections [9]. Primary ciliary dyskinesia is found in a subset of heterotaxy patients, but the exact prevalence remains unknown. The co-occurrence is also attributed to the presence of dynein gene defects found in both [10]. The repeated left-sided aspiration pneumonia can also be associated with left-sided vertical bronchus as found radiographically. Due to the ambiguity from the co-existing impaired immune response and defective ciliary clearance, it remains a possibility that cannot be confirmed.

Etiology and pathophysiology

The etiology and pathophysiology in the development of HS remain ambiguous and unclear. Heterotaxy in individuals is found in isolation or in combination with other features as a part of genetic syndromes such as Kartagener syndrome. 

The major cause entails the vast variety of rare genetic mutations associated with environmental factors affecting embryological development during pregnancy. These include:

1. Genetic mutations involving ZIC3, NODAL, CFC1, ACVR2B, LEFTY2, CITED2, and GDF1 [11], with the mode of inheritance involving autosomal-dominant pattern with incomplete inheritance, autosomal-recessive, and, in rare cases, X-linked patterns. These are the genes that have been identified in encoding proteins of the transforming growth factor (TGF)-beta pathway. 

2. Environmental factors like maternal diabetes, family history, especially in male infants, retinoic acid use, and maternal cocaine use during the first trimester. They are responsible for sporadic cases [12].

The pathophysiology of heterotaxy is explained by the molecular and cellular mechanisms involved in normal left-right asymmetry. Despite extensive research, a comprehensive understanding of the exact mechanism remains unclear. 

Studies on animals such as mice have demonstrated that the left-right axis is determined by ciliary movement located in the primitive node. Monocilia in nodes consists of motile and non-motile cilia. Motile cilia, composed of left-right dynein in mice and DNAH5 in humans (responsible for Kartagener syndrome), is located in the center of the node compared to non-motile mechanosensory cilia, expressing calcium ion channel polycystin-2, Pkd2, found in the peripheral region of the node. The clockwise rotation of motile cilia generates a leftward nodal flow, which produces the initial break in the body symmetry [13]. This results in an increased influx of Ca^2+^ ions in cilia through Pkd2, which causes the activation of the NODAL gene in the left side of perinodal cells of the primitive streak, transferring of these asymmetric signals to the lateral plate of mesoderm, and expression of gene products including PITX2, LEFTY1, and LEFTY2. The TGFs are involved in the process; growth differentiation factor 1 (GDF1) is important for transporting NODAL; the bone morphogenic protein (BMP) negatively regulates the Nodal expression, and LEFTY1 in the left and LEFTY2 in the center antagonize to restrict the NODAL signaling mechanisms. PITX2 is the laterality gene expressed in the lateral mesoderm plate for asymmetric organogenesis of the heart and other viscera. Any mutation in PITX2 causing its dysfunction produces cardiac malformations [14]. In conclusion, leftward nodal flow generates asymmetry signals and activates the left-sided specific growth and transcription factors to form left-sided viscera from the left lateral mesoderm plate and right-sided structures are developed on the right lateral plate by default.

Hence, left isomerism results from the randomized distribution of left determinants producing an abnormal nodal flow. Thus, the left signal is activated on both sides. Right isomerism occurs when left determinants are insufficient, and the left signal is not activated on both sides. Situs inversus, however, is formed when there is inverse nodal flow.

Clinical features

The abnormal lateralization of visceral structures in the body develops a clinical presentation that ranges from minimal disease symptoms to complex cardiovascular structural defects that can prove fatal. It is associated with a wide array of extracardiac manifestations. Autopsy studies have demonstrated the involvement of various systems in 40-70% of cases [15].

LAI refers to the similar appearance of the structures on both sides of the body to the ones that develop normally on the left side. The atrial appendages on both sides resemble the left atrium, and the term “isomeric atria” is used for both. As a result, both atria receive the pulmonary veins. It is associated with interrupted inferior vena cava with the azygos or hemiazygos continuation of inferior vena cava (incidence is about 80%). The aberrant right subclavian artery is another feature and can result in the compression of the esophagus causing dysphagia and can develop aneurysmal dilatation called diverticulum of Kommerell. In contrast to right isomerism, cardiac problems are milder. Among the prevalent cardiac diseases, studies have revealed that two-thirds of problems in left isomerism are manageable with biventricular repair. The remaining presents with univentricular atrioventricular connection [1]. The sinus node, normally located in the right atrium, is absent, leading to sinus nodal dysfunction and frequent episodes of atrial flutter and fibrillation. Such patients are more susceptible to conduction defects like atrioventricular block, intraventricular conduction delay, and atrioventricular nodal reentry tachycardia [16,17]. Moreover, due to a concordance between cardiac and pulmonary defects, LAI has bilaterally symmetrical bilobed lungs and hyperatrial bronchi. Hyperatrial bronchi refer to bronchus originating and coursing inferior to the pulmonary artery.

Extracardiac manifestations are various and more common in LAI. Polysplenia is usual. Similar to asplenia, it is hypofunctioning and responsible for producing a weak immune response. It is the quality of function that is relevant rather than the quantity of spleen. However, studies on this are scarce, and no definite evidence is available. The risks of infection may be decreased than in asplenia; still, it can result in lethal sepsis [4]. In most cases, the liver is midline and transverse. In such patients, the presence of jaundice must raise suspicion for biliary atresia, which occurs in 10% of cases [18]. The stomach has variable locations and is usually found on the right side. Tracheoesophageal fistula, duodenal atresia, annular pancreas, and anal atresia (in atresia) are other abnormalities of the gastrointestinal tract. Intestinal malrotation with or without volvulus is a widely prevalent finding in LAI, and some form of it is found in around 70% of cases [4]. The capricious structural defects in the gastrointestinal tract generate atypical chest pains, aspiration pneumonia, and recurring intestinal obstruction. Genitourinary defects that result include horse-shoe shaped, hypoplastic, dysplastic, or absent kidney and urinary tract abnormalities, predisposing to recurrent infections, hydronephrosis, and deranged renal function.

Investigations and management

Any patient presenting in the ER with fever requires a complete blood count with differentials, C-reactive protein, and blood culture. The most significant investigation in patients presenting with infections and HS including polysplenia should involve assessing the functioning of the spleen. This can help in identifying the bacterial source behind the bacteremia and deciding the type and duration of antibiotics for the management. Ultrasound or CT scan cannot be utilized solely to assess splenic functioning. Some other tests should be employed, which could have been beneficial in our patient. These include the detection of Howell-Jolly bodies in the peripheral blood smear by light microscopy or quantification via flow cytometry. ^99m^Technetium-labeled scintigraphy or pitted erythrocyte count measures the erythrocytes not cleared by the spleen. Thus, the percentage of Howell-Jolly bodies in the blood quantifies the degree of impairment of the spleen and can be a pivotal test for defining the severity and recurrence of the disease, ultimately helping to decide on the duration and prophylaxis related to antibiotics.

In addition, some other tests such as ECG, transthoracic echocardiography, cardiac catheterization, chest X-ray, abdominal ultrasound, upper gastrointestinal contrast studies, and CT/MRI scan should be done in all newly diagnosed HS patients after the discharge. Angiography is challenging to perform in LAI; thus, a percutaneous approach through neck veins is used. Detailed family history, along with genetic testing, may be required in a few cases. The routine prenatal screening ultrasonography and fetal echocardiography form the hallmarks for the prenatal diagnosis. Fetal MRI may be required in some cases.

In the case of hypofunctioning spleen, appropriate and timely immunization and antibiotic prophylaxis are crucial. All the data regarding it stems from the sickle cell disease (SCD) population. Therefore, all patients are recommended prophylactic antibiotic treatment with penicillins from the time of diagnosis until they reach at least five years of age. For adults, lifelong antibiotic therapy is recommended after sepsis or severe infections. Moreover, immunization against all encapsulated organisms such as pneumococcal, meningococcal, and *Haemophilus influenzae* should be done. Seasonal influenza vaccination is also mandated. The role of prophylactic antibiotics in asplenia is firmly established and follows the guidelines for SCD. The increased risk of bacteremia with polysplenia, though present, is not highly prevalent. There are no studies that have evaluated the risk amelioration of bacterial infections in the polysplenia population. Therefore, antibiotic prophylaxis is not routinely recommended [6].

Early diagnosis and recognition of abnormalities are essential for the effective management and prevention of complications in all HS patients. Therefore, other complications should be evaluated for management as well. Surgical correction is the fundamental step in managing HS, though, in LAI, anatomic disturbances can present a hurdle. In biliary atresia, repair by Roux-en-Y Kasai operation or a liver transplant may be required. Intestinal malrotation requiring a pre-emptive laparoscopic LADD procedure remains controversial; however, since emergency laparotomy is associated with significant morbidity, offering the procedure to those with malrotation remains reasonable [19,20]. Biventricular repair is done for cardiac abnormalities, while the Fontan procedure is performed in case of univentricular cardiac defects [21]. Most procedures are palliative in nature, as normal anatomy cannot be fully and successfully restored by surgical procedures.

Prognosis

The prognosis in LAI is greatly influenced by the type and number of associated cardiac problems, and in the absence of such defects, it is quite promising. The mortality is approximately 50% in LAI, mostly attributed to the presence of heart block [22]. Bacterial sepsis in HS is associated with a mortality rate of 44% [23]. Prenatal diagnosis is especially useful in asplenia patients as around 10% of cases can lead to stillbirths as compared to 6% in polysplenia (where the main cause remains bradyarrhythmias). One study has demonstrated that the postnatal death rate in live-borns asplenia babies is 43% vs. only 13% in polysplenia babies [24]. The major cause in both remained cardiac. The morbidity is significantly high owing to the propensity for several complications together with deranged anatomy. This disease confers a dilemma in both its diagnosis and management and remains a challenging condition to be dealt with.

## Conclusions

We discussed an unusual presentation that led to the diagnosis of a rare underlying condition termed HS/left isomerism. Hyposplenia can occur in HS patients regardless of spleen anatomy or presence. The bacteremia and recurrent pneumonia associated with polysplenia in such patients raise questions regarding prophylactic antibiotics and immunization similar to asplenia, which require further studies involving measures such as the quantification of splenic function. More studies to determine the specific immunological disruptions occurring in HS are also required. The question of whether the guidelines for the management of hyposplenism in polysplenic HS should be similar to those for non-heterotaxic hyposplenism and heterotaxic asplenic patients is pertinent.

This condition can have variable presentations and remain undiagnosed for a long time unless congenital heart defects are severe enough to warrant at-birth investigations. This adds to the morbidity and mortality associated with it. To prevent this, early diagnosis and management are essential. In cases of high suspicion and those with a family history, early CT scan and echocardiography can provide timely treatment and prevention of complications. A multidisciplinary team consisting of internists, gastroenterologists, cardiologists, radiologists, surgeons, and nurses is essential to provide timely and imminent care.
